# Can Changes in Financial Performance Be Used in the Diagnosis of Neurocognitive Disorders? A Systematic Review of Findings from Greece

**DOI:** 10.3390/brainsci14111113

**Published:** 2024-11-01

**Authors:** Vaitsa Giannouli

**Affiliations:** School of Medicine, Aristotle University of Thessaloniki, 54124 Thessaloniki, Greece; giannouliv@hotmail.com

**Keywords:** financial incapacity, financial abuse, old age, forensic neuropsychology, Greece

## Abstract

**Background/Objectives:** Elder abuse, and more specifically financial exploitation, is expected to be a major problem in modern societies as the worldwide population is getting older. Neuropsychological protocols regarding financial capacity assessment are the only available window allowing us to view the cognitive–emotional–behavioral strengths/deficits and vulnerabilities of individuals. Given the paucity of relevant research in Greece in the most vulnerable individuals such as older adults suffering from neurocognitive disorders (NCDs), this systematic review attempts to investigate whether NCDs impair financial capacity and to highlight the most important factors that can predict financial incapacity in Greek older patients and the likelihood of financial abuse. **Methods:** A systematic search was conducted in Embase, PsycINFO, and PubMed. **Results:** The search identified *n* = 21 relevant research articles. The synthesis of available evidence supports that financial incapacity is clearly demonstrated in the group of Greek older adults suffering from NCDs of different severity and etiology; thus, such changes can assist diagnosis, treatment, and care of these individuals, but the implications for elder abuse in the Greek cultural context have not been examined in detail so far. **Conclusions:** Given the unique source of information that neuropsychological assessments represent by revealing the importance of factors such as arithmetic cognition and relevant brain volume changes in the left angular gyrus, depressive mood, apathy, frailty, vascular risk factors, and financial illiteracy, forensic neuropsychology can play a vital role in protecting older individuals from financial abuse.

## 1. Introduction

Abuse can take various forms, but it is still difficult to detect cases of abuse in older adults given the plethora of definitions. Among the suggested forms of abuse, seven are examined in relevant empirical research, such as physical abuse, psychological/emotional abuse, sexual abuse, neglect, self-neglect, abandonment, and financial exploitation [1,2]. Without first developing common definition(s) across countries, scarce research is examining a specific form of elder abuse, that is, elder financial abuse [1]. One of the most cited definitions is the one according to the National Center on Elder Abuse (2005), which defines financial abuse as the ‘illegal or improper use of an elder’s funds, property, and/or assets’, a definition that implies that intentional actions of financial abuse occur when lack of independence characterizes the individual/victim. Diminished financial knowledge, skills, and emotional vulnerability/susceptibility can lead an older adult to experience financial exploitation [2]. These individual characteristics constitute financial capacity, which in its turn substantially influences the incidence of financial abuse.

Financial capacity in the literature has been approached in several ways: as an instrumental ability of daily living (IADL) relating solely to simple money management, as a group of skills relevant to independence (including basic and complex skills, financial execution, and judgment), as a multidomain concept that can take the form of neuropsychological testing of cognitive domains such as procedural knowledge, semantic and declarative memory, and executive functions, among others, and/or as a solely decision-making process with relevant steps that the individual follows successfully or not [2]. The various definitions of financial capacity are a source of problems for healthcare professionals as individual characteristics (e.g., demographics such as age, marital status, family network), socioeconomic variables (such as education, income, occupational background, financial literacy), health status (physical problems such as disability, comorbidities, and/or mental problems such as depression and loneliness), and cognitive status/abilities (which are highly affected by the existence of NCDs) affect in varying ways and degrees what is considered to be financial capacity in the above definitions [2]. From these numerous variables, neuropsychologists should focus on the relationship between cognitive impairment and financial abilities and particularly on specific cognitive domains which are vital for financial decision making and personal financial management (such as attention, which is the ability to selectively attend to specific information whilst ignoring irrelevant information) [2]. Emotional changes and mood disorders such as depression (which has been claimed to negatively influence not only general cognitive performance across the lifespan but also has a negative impact on specific cognitive domains in old age such as attention) are also of interest, as depression may influence cognition and (this in turn may influence) financial capacities in particular [for a relevant review of recent studies, see 2]. Furthermore, neuropsychologists should also focus on the brain underpinnings (e.g., angular gyrus) of numerical and mathematical processing, which are linked to financial capacity, and on the role of vascular changes (e.g., hypertension, hypotension, dyslipidemia, diabetes) and diseases (e.g., carotid stenosis, history of coronary artery disease, transient cerebral ischemia, cardiac arrhythmia) on financial abilities [for a relevant review of recent studies, see 2].

Additionally, research in victimology discusses the cognitive, emotional, and behavioral vulnerabilities appearing in old age in healthy as well as in older adults with a medical diagnosis [2], but there seems to be a clear cultural difference in what is considered/perceived to exactly fit the term ‘financial abuse’, even in Western countries (such as the U.S.A. and Greece) [3,4].

For example, in Greece financial abuse is reported to be a taboo topic (with negative connotations), which stigmatizes the family members and diminishes the independence of the older adult(s) [5]. This may lead to underreporting, underinvestigating, and underprosecuting cases of financial abuse of older adults [6] as has been found in interviews conducted with residents of urban and rural areas in Greece, who disregard and/or avoid mentioning financial abuse among the types of elder maltreatment [7,8]. Elder financial abuse can be regarded both as a form of family violence (when the perpetrators are the relatives of the older victim) or by a third person [9], while overall country-specific socioeconomic inequalities can influence the prevalence and experience of elder financial abuse by the societies and the older adults themselves, thus creating a mosaic of varying perspectives even among European countries [10,11,12,13,14].

The above create uncertainty to healthcare professionals (neuro/geropsychologists, geriatricians, psychiatrists, neurologists, social workers, interdisciplinary teams, etc.) and legal experts (barristers, judges) as well as policymakers and laypeople regarding what is financial elder abuse, how to measure it, and what are its predictors in an era of multiple changes such as pandemics [15] and technological advancements, such as artificial intelligence (AI) [16]. As a result, financial decisions made by older adults (with and without a diagnosis of neurocognitive disorder), such as making a significant purchase, giving a large financial gift, allowing someone to access their personal accounts, and/or having someone take over finances and management of funds, among other decisions, are approached with uncertainty by professionals regarding the method(s) that they should follow in order to decide on the competence and the independence of the individual who made the decision(s).

Thus, the wider aim of this systematic review is to provide not just an up-to-date examination of the existing tools and procedures in Greece for the objective assessment of financial capacity/incapacity and the vulnerabilities that may characterize an individual and make them prone to abuse [17] but to examine what the current assessment protocols in Greece reveal about the cognitive, emotional, and behavioral deficits of older patients and the importance of these deficits in the clinical diagnosis and prediction of elder abuse. More specifically, this review aims to explore whether neuropsychological disorders impair financial capacity in Greek older patients and whether this can impact financial abuse.

## 2. Materials and Methods

**Figure 1:**
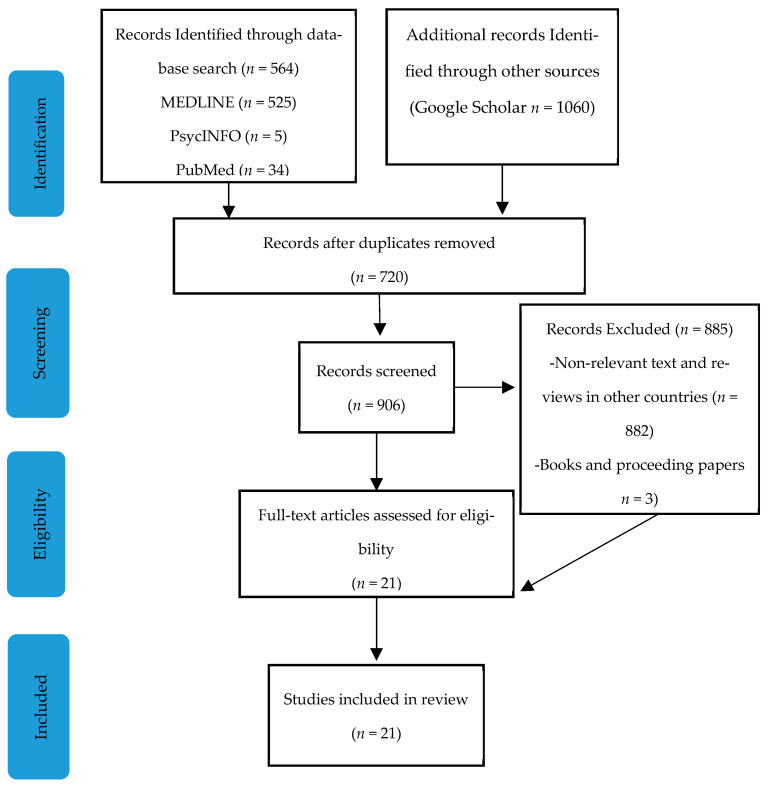
Flow chart illustrating the search–identification strategy for included articles.

A systematic search in Embase, PsycINFO, and PubMed identified *n* = 21 relevant research articles from December 2023 to March 2024. The electronic search was initially performed using the following terms: ‘financial capacity’, ‘Greece’, ‘older adults’, ‘patients’, ‘neurocognitive’, ‘disorder’, ‘healthy’, and ‘abuse’. The extensive search was filtered for records published after 2000, and only peer-reviewed papers published in journals with official impact factors (IF) were examined for inclusion. All records were screened by title and abstract, and if judged as possibly eligible they were examined on a full-text basis by the researcher. In addition, the reference lists of the included articles were manually cross-referenced in order to identify any additional relevant articles. The inclusion criteria for the studies were as follows: published after 2010, being primary articles published only in journals with peer reviewers, examining healthy as well as older patients over the age of 65 years old, with or without a diagnosis of a medical disorder/NCD, and measuring financial capacity (not testamentary capacity) only in the Greek language/cultural context. The exclusion criteria were as follows: studies not published in English journals with IF and reviewers and those studies that did not meet the other inclusion criteria. A meta-analysis was not performed due to the heterogeneity of the studies (see Figure 1).

## 3. Results

Reviewing the previous literature provides a way to find the factors that can influence financial incapacity and subsequently the probability of abuse. More specifically, only one relevant standardized test exists in Greece for the assessment of financial (in)capacity in older adults, which follows Marson’s theoretical framework about the importance of cognitive factors on financial decision making [18]. The Legal Capacity for Property Law Transactions Assessment Scale (LCPLTAS) [19] is a Greek adaptation of the Financial Capacity Instrument (FCI) used in the U.S.A. Marson’s conceptual framework highlights three element levels for financial capacity: (1) specific financial abilities, (2) broad domains of financial activity, and (3) overall financial ability [18]. Therefore, the LCPLTAS consists of seven domains, each measuring relevant skills linked to financial affairs, such as basic monetary skills, cash transactions, bank statement management, bill payment, financial conceptual knowledge, financial decision making, and knowledge of personal assets [19]. These domains are the same as those included in the FCI, with the exception of the domain which examines checkbook management, which is considered as not applicable in the Greek cultural context, as checkbooks are not used by individuals.

Among the studies analyzed using this test, all found severe cognitive deficits in Greek older adult patients regardless of their demographic characteristics (age, gender, marital status, education, geographic region) [19]. Financial incapacity seems to be based not only on the performance on the LCPLTAS but also on older adults’ neuropsychological performance in basic neuropsychological tests measuring attention (Trail Making Part B), overall cognition (Mini Mental State Examination (MMSE)), and depression (as measured with the Geriatric Depression Scale (GDS)), three tools that are in use in everyday clinical practice in Greek state and private healthcare settings and which appear as strong predictors of incapacity and subsequently of vulnerability to abuse.

The use of LCPLTAS has indicated that in patients with Alzheimer’s disease (AD) of all stages (mild, moderate, and severe), there are objective deficits in all seven financial domains [20], thus creating multiple domains in which financial abuse can occur. These deficits co-occur with difficulties in simple everyday arithmetic tasks [21], thus raising the importance of cognitive factors and brain health, but no connections are found with the experience of stressful life events and experienced emotional stress [22]. These objective deficits are not accompanied by accurate estimations made by family members/caregivers [23], a finding that raises our awareness over the utility and use of third-party estimations/perceptions in the Greek cultural context and courts. This is also confirmed for the group of older patients with a diagnosis of frontotemporal dementia (FTD) and their caregivers’ overestimations, even though no evident reason for distorting reality is present [24]. Given the lack of self-awareness not only of general cognitive abilities but also of more focused financial abilities, which is a common symptom across NCDs [25], it is clear that objective neuropsychological assessment of cognitive capacities/abilities is necessary, as self-reports or estimations by others may not reveal the truth, while the older patients (with no motivation for malingering) are not aware of these deficits.

Another important finding is the negative influence of depressive symptomatology not only on AD patients’ financial abilities [26] but also on Parkinson’s disease (PD) [27] and mixed dementia (MD) [28], as well as mild cognitive impairment (MCI) patients’ performance [29]. More specifically in MCI patients, the existence of vascular risk factors predicts poorer financial abilities [30], while new data can inform neurolaw, as diminishing brain volumes in the areas of the left angular gyrus (which is associated with arithmetic and mathematical processing) and the amygdala (which is involved in the regulation of emotions) are strongly correlated to changes in diminished financial capacity performance over time [31].

Financial literacy has also been found to play the role of a catalyst for financial capacity deficits in NDCs, as MCI patients with higher education show significantly less problems and vulnerability to financial spam in comparison to illiterate MCI patients and controls with low education [32]. In addition, behavioral variables such as sleep problems in MCI and AD patients may be a useful sign of deficits in financial cognition [33] as well as non-justified altruistic decisions which also indicate financial incapacity in older adults with different diagnoses of NCDs [34]. Surprisingly, genetic variables such as the APOE e4 gene (which increases the risk of developing AD and therefore cognitive deficits) does not seem to differentiate individuals regarding their financial capacity deficits [35], while data on beta-amyloid and tau for AD come only from a small-scale study of Greek AD patients and show no relationship between financial deficits and these biomarkers [36]. Furthermore, research for financial capacity performance and a-synuclein for Parkinson’s disease and NfL for neurodegeneration does not exist. Finally, two additional psychological factors to take into consideration in the neuropsychological assessment of financial capacity, especially in FTD and PD, is the existence of apathy, which seems to have a devastating influence on financial capacity performance, even greater than depressive symptomatology [37], while higher frailty, especially in Greek older community-dwelling women, predicts perceived financial exploitation [38] (see Table 1).

## 4. Discussion

Given that financial abuse and financial capacity/ability/competency are entwined, the above findings are of extreme importance as they can predict who is vulnerable as a future victim of financial abuse. What is of importance is that financial impairments could be a sign for further clinical investigation, as in Greece the majority of older adults remain undiagnosed due to limited access to healthcare services or due to the stigma of a dementia diagnosis. But still, a question remains: how does a neuropsychologist/geropsychologist/geropsychiatrist dive into financial matters of older adults? Despite the urgent need for standard widespread routine assessment protocols for financial capacity and financial abuse to be used in different countries, there is no formally applied assessment or guideline(s) in Greece, but there does not exist a shared procedure in Europe or in the U.S.A. either [40]. A robust finding from the abovementioned relevant research in Greece which may be of interest both to researchers as well as policymakers is that a plethora of cognitive deficits and emotional vulnerabilities are found in the majority of older adults with different types of NCDs, and these identified factors need to be examined or considered as a source of financial vulnerability. This renders a large proportion of the older Greek population unprotected as a big portion of Greek older adults after COVID-19 has been examined and given a relevant diagnosis [41]. Given the complete lack of estate planning and advance care planning (e.g., presence of a valid will, a durable power of attorney for healthcare, and/or living will) in the Greek older population [42], the above findings can be a source for professionals identifying who cannot handle their finances and may also assist in determining competency, issues of responsibility for financial crimes, and in sentencing/mitigation in litigation.

Although the existing approach in theory and practice in Greece is based on an instrument that presents neutral and/or hypothetical stimuli, and not stimuli that examine the actual situation for which a specific financial judgment and/or transaction has to be made, and in addition to that this instrument does not include contextual factors, such as perceived financial, psychological, and relationship insecurity around personal finance which is correlated to cases of actual financial abuse [43], new efforts of standardization in Greece of relevant instruments from abroad, specifically designed for the assessment of older adults for forensic purposes, are underway.

This new alternative conceptual model does not only take into account the components of cognitive ability [e.g., crystallized intelligence (vocabulary and financial facts as well as relevant procedural knowledge) and fluid intelligence [memory, reaction speed, and problem solving and planning]) that are necessary for an efficient management of financial issues, but it examines the intersection of contextual factors (financial situational awareness, psychological vulnerability, and susceptibility), intellectual factors (related to the expression of choice, rationale, understanding, and appreciation of a financial decision), and consistency with values, and based on these components the model predicts the integrity of financial decisional ability (capacity) of an older individual [43].

For example, such initiatives of standardization of easy-to-use and to score tools include questionnaires such as the Lichtenberg Financial Decision Rating Scale (LFDRS) [44], the Financial Decision Tracker [45], the Financial Vulnerability Assessment [46], and the Family and Friends Interview [47], so a more complete assessment can be provided, which will include and combine both perceived as well as actual performance of individuals. Such a person-centered approach to assessment may allow healthcare professionals to enhance autonomy, where possible, by targeting modifiable habits and behaviors [48]. This implies that even in the context of NCDs and/or other mental disorders with functioning impairments, the older individual may still possess important areas of reserve/strength, such as financial judgment [48]. These results obtained by protocols in the American older population (e.g., by administering LFDRS, FDT, FVI) [44,45,46,47] do not emphasize so much the cognitive deficits as found in the abovementioned Greek studies in similar older populations, especially the incompetence demonstrated in the AD groups (of different levels of severity). However, the findings of LCPLTAS corroborate the cognitively focused impairments coming from FCI administration in the U.S.A. across the dementia spectrum (e.g., for groups such as mild, moderate, and severe AD, MCI, Parkinson’s disease) [18,49]. It is also interesting that results from other European countries in older adults are scarce. For example, one study from Holland suggests that in the earlier stages of PD, when cognitive impairments are relatively mild, some problems may be observed in financial capacity, yet other domains of financial capability appear less affected [50]. This is not in contrast with Greek findings, as the participants did not have a diagnosis of dementia but only of Parkinson’s disease. Additionally, findings from Israel [51] are in line with findings from Greece about the predictive role of unjustified financial altruism as an important variable for relevant deficits and as a sensitive behavioral marker for early Alzheimer’s disease. Finally, a study from Italy provides only preliminary results in MCI with a relevant tool which builds on the conceptual model of FCI, showing problems in all financial domains but with the exception of ‘reading abilities’, ‘purchase’, and ‘financial judgments’ [52]. A point that could be concluded from the included Greek studies is that regardless of the diagnosis, the specific cognitive patterns of cognitive deficits affecting patients with different pathologies are not of importance to financial capacity performance but MMSE; therefore, just the level of general cognition plays a pivotal role in predicting overall deficits in all financial capacity domains (such as basic monetary skills, cash transactions, bank statement management, bill payment, financial conceptual knowledge, financial decision making, and knowledge of personal assets), as they are homogeneously impaired [19].

At the same time existing findings, despite the plethora of different instruments in use from abroad [53], should be used and assist professionals as well as policymakers in designing prevention and protection programs for older adults who are vulnerable and may end up as victims of financial abuse. For example, with respect to neurodegenerative disorders, it would be interesting based on the specific financial ability deficits and depending on the cognitive status characterizing these disorders (e.g., in patients with Parkinson’s disease or patients with AD) to take into account these deficits and improve them in order to reduce fraud risks in light of cognitive rehabilitation programs tailored to patients′ specific impairments [54,55]. In the case of Greek older patients specifically for Parkinson’s disease, a proposal targeting apathy could be implemented [37], while for AD patients cognitive rehabilitation focusing on attention-executive function at all stages of the disease could be designed [19] and/or specific domains could be addressed as research shows that they are affected negatively in time with a specific order starting with cash transactions, bank statement management, and financial decision making [56].Three final points to keep in mind are focused on the barriers relating to the ways that the legal systems function in different European countries including Greece which are not the same as in the U.S.A. system [57]. The legal differences pose significant hurdles on intercultural dissemination of existing knowledge. This implies the use of relevant financial capacity and financial elder abuse tests (mainly coming from the U.S.A. must be adjusted so researchers and clinicians adopt and adapt them to the reality, e.g., linguistic, educational, financial, social, etc.) of the country in which they are intended to be used. The second point to consider are the cross-cultural aspects regarding the presence, magnitude, etiology, as well as the terminology, meaning, interpretations, and implications of culture-related differences imposed on cognitive test performance of individuals [58,59,60,61]. It is worth mentioning here that even the labeling of the severity of specific forms of financial elder abuse may differ in different countries, as findings in the U.S.A. put an emphasis on theft and scams, financial victimization, financial entitlement, coercion, and signs of possible financial exploitation, followed last by money management difficulties [62], something that is not corroborated by research in Greece [5]. Although currently no data from Greece exist for minority group members, increased self-reflection about multicultural awareness is a third point for further consideration and research. This last point makes it necessary to consider the importance of understanding and incorporating financial assessment of the characteristics of members of minority groups, which so far have been largely disregarded in various countries, and their participation in relevant research is severely limited [63,64].

One of the major limitations of this study is that in Greece there is only one group of researchers/clinicians (located in Northern Greece) conducting such research despite this very important clinical and practical problem. Based on this, some questions arise: (1) Why are there not more groups researching in this line of work? One possible answer could be that ‘financial abilities’, which is a broader term than ‘testamentary capacity’, are not mentioned in legal texts, and clinicians are not properly educated on their importance. (2) What tools and methods also exist to assess changes in financial performance in Greece? There are a few standardized neuropsychological tests in use in Greece specifically designed for older adults with relevant norms, and they mainly focus on general cognition (e.g., MMSE—only one numeracy item) rather than on specific financial skills, while very few tests examine arithmetic skills as a substitute for financial capacity (e.g., the Math4Speed Test, which measures only simple and complex arithmetic skills [65], and the Wechsler Adult Intelligence Scale-4th Edition (WAIS-IV GR) subtests: arithmetic [66]). (3) What barriers exist in implementing the measurement of financial capacity in other centers in Greece? One of the major barriers is the limited knowledge of clinicians and researchers about established tools in the Greek population and the lack of co-operation. (4) What roles are care providers expected to take on when detecting the problem? Attempts have also been made to propose a framework about ‘what to do’ after the diagnosis of the financial capacity deficits such as training of the family members and caregivers on how to support decision making and/or when to intervene [15], but so far there is no official legal framework that applies for all such diagnoses across Greece except for the paternalistic legal role of ‘guardians’, who are persons who have the legal authority to manage the personal activities and/or resources of the older patient who is unable to make financial decisions.

Despite the above, recent research in other countries shows that psychosocial, socioeconomic, and sociocultural aspects can influence the cognitive functioning in the population of community-dwelling older adults [67], and at the same time cognitive ability per se seems to be an important predictor of debt burdens in older adults from the general population, as findings for older individuals with higher cognitive ability show that they take on higher debt levels relative to their counterparts in complex financial environments [68]. In addition, for relevant constructs to financial capacity such as financial literacy (which represents just the knowledge of financial concepts and skills), findings from abroad (mainly the U.S.A.) support that lower literacy in community-dwelling older adults is linked with mortality [69], and the inevitable declining literacy (not only financial but also health literacy) in normal aging is related to poorer decision making, greater scam susceptibility, and lower wellbeing for the older persons [70]. Furthermore, loneliness for these individuals compromises healthcare and financial decision making, especially for those with lower global cognition and, more specifically, for those scoring lower in working memory tasks [71], while limbic structures influencing motivational and emotional processes associated with financial decision making have been found to also be of interest in normal and not only pathological aging [72]. The above findings concerning community-dwelling older adults show that there should be a wider debate not only for the patient groups, considering the fact that financial literacy and exploitation happen in person as well as online [73], making not only the assessment but also relevant interventions even more complex.

## 5. Conclusions

Research in the past decade has documented that elder financial abuse is a major multi-dimensional problem and that we must increase our efforts to reduce financial exploitation, but little research so far examines this topic in Greece and the cognitive underpinnings or other factors which render someone more vulnerable as a victim of financial abuse have been little investigated. Despite being poorly defined, two conceptual models/frameworks focus on financial capacity (Marson’s and Lichtenberg’s approaches). The two approaches are not mutually exclusive, but they initiate an important dialogue on how they can be combined and can assist in examining financial elder abuse with both a standardized assessment method and a person-centered approach/orientation.

**Figure 2:**
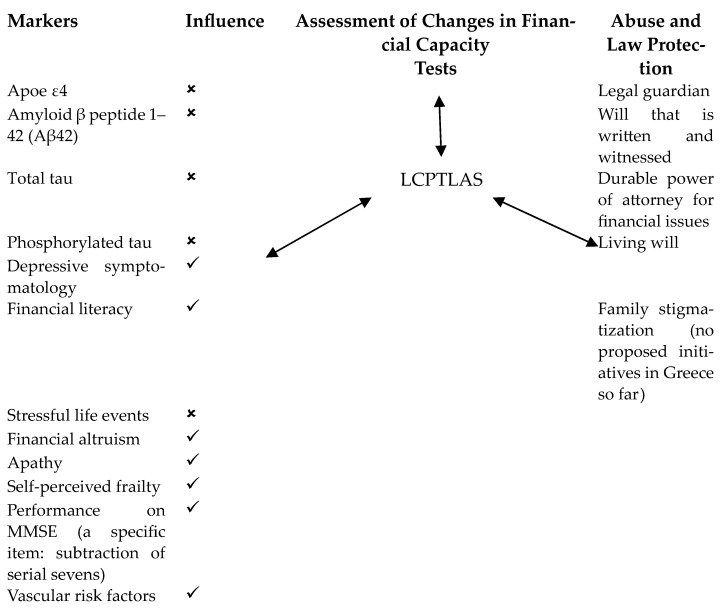
Scheme for the assessment of financial capacity performance of older people. ✓ Linked; 🗴 Not linked.

This review of recent developments in Greece consolidates existing knowledge on the significance of detecting financial capacity deficits in old age by highlighting the role of variables found to be of importance in empirical research such as arithmetic cognition and relevant brain volume changes in the left angular gyrus, depressive mood, apathy, frailty, and financial illiteracy in Greek older adults (see Figure 2). This knowledge may allow us not only to provide appropriate assessment by adding new reliable and valid tools for use but will help us incorporate these variables/factors that can predict financial abuse and therefore organize the protection of older individuals suffering from an NCD through therapy interventions and training of family members/caregivers/the public and the older adults themselves over this topic.

## Figures and Tables

**Table 1:** Studies from Greece showing deficits in financial capacity in older patients with NCDs.

Studies	Greek Older Participants	Financial Capacity Assessment Tools	Aims	Results	Negative Influence of NCD(s) on Financial Capacity
Giannouli et al., 2018 [19]	719 participants: patients with aMCI, mild, moderate, and severe AD, mild PD, mild VD, moderate FTD, moderate MD, and healthy controls (HCs). All without depressive symptomatology.	LCPLTAS	To examine whether different brain pathologies can influence financial capacity.	Financial capacity deficits in all patient groups.	Yes
Giannouli et al., 2022 [20]	109 patients diagnosed with mild AD.	LCPLTAS	To examine which scores on classic neuropsychological tests predict possible financial capacity deficits.	MMSE score in a specific item: subtraction of serial sevens predicts deficits in LCPTLAS.	Yes
Giannouli et al., 2023 [21]	110 patients with an AD diagnosis, 107 patients with a diagnosis of mild MCI, 109 HCs, and 94 PD patients.	LCPLTAS	To examine if arithmetic errors have an influence on financial capacity.	Overall, the results reveal that all older patients did commit arithmetic errors that influence financial capacity performance.	Yes
Giannouli et al., 2023 [22]	122 AD patients and 146 HCs with similar demographics.	LCPLTAS	To examine the influence of stressful life events on financial capacity.	Stressful life events do not influence financial capacity and relevant vulnerability to financial exploitation for HCs and AD patients.	Yes
Giannouli et al., 2022 [23]	109 mild AD patients.	LCPLTAS	To examine the connection between biological factors (sex), social determinant (SES), behavioral, and psychological symptoms of dementia (BPSD) measured with neuropsychiatric inventory (NPI) scores with financial capacity.	BPSD (measured with NPI) was found to negatively correlate with estimates of financial capacity.	Yes
Giannouli et al., 2022 [24]	28 patients with a diagnosis of FTD (following the International Consensus Criteria for bvFTD-FTDC) and 28 HCs. Both groups did not suffer from depression or other mood disorders. Caregiver estimations were also recorded.	LCPLTAS	To examine financial capacity in FTD patients and to investigate the perceptions of their caregivers.	Financial capacity in FTD patients is severely impaired compared to controls, but caregivers of FTD patients tend to overestimate the patients’ financial performance.	Yes
Giannouli et al., 2022 [25]	35 mild AD patients, 41 single-domain aMCI patients, 41 multiple-domain aMCI patients, and 30 HCs.	LCPLTAS	To examine the accuracy of self-estimations for financial capacity.	Financial capacity performance is overestimated in MCI and mild AD patients.	Yes
Giannouli et al., 2021 [26]	109 participants were divided into 4 groups: mild AD with and without depressive symptoms and cognitive normal HCs with and without depression.	LCPLTAS	To examine whether comorbid depression can influence financial capacity in AD.	Financial capacity in mild AD patients is severely impaired when depression co-exists, compared to mild AD without depression or in depressed older adults without NCDs.	Yes
Giannouli et al., 2019 [27]	60 participants divided into four groups (PD with and without depressive symptoms, non-demented elders with and without depression).	LCPLTAS	To examine the influence of depression on financial capacity in PD patients.	PD patients’ performance in cognitive functioning and financial capacity is severely impaired, while there is a statistically significant difference between depressed and non-depressed PD patients.	Yes
Giannouli et al., 2023 [28]	115 participants divided into four groups: MD patients with and without depressive symptoms and HCs without depression as well as older adults suffering from depression.	LCPLTAS	To examine the influence of depression in MD, that is, in cases where there is the co-existence of AD and VaD.	Financial capacity in MD patients is severely impaired when depression co-exists compared to suffering only from depression and HCs.	Yes
Giannouli et al., 2022 [29]	120 multiple-domain aMCI with stable and increased levels of depression at one year, aMCI without depressive symptoms, and cognitively intact elders with and without depression.	LCPLTAS	To examine if longitudinal (1-year) changes in cognition in aMCI can predict financial capacity deficits expressed over time.	Multiple-domain aMCI patients’ performance regarding financial capacity is severely impaired when depression co-exists, resembling the performance of patients with mild AD, and further decline is found when depression deteriorates.	Yes
Giannouli et al., 2022 [30]	112 participants were divided into three groups: patients with single-domain aMCI, patients with multiple-domain aMCI, and HCs, while taking into consideration whether participants had a diagnosis of one vascular risk factor (VRF) or disease.	LCPLTAS	To examine the role of VRFs on financial capacity.	A larger vascular burden in aMCI is correlated with lower financial capacity.	Yes
Giannouli et al., 2019 [31]	15 aMCI patients were examined 3 times during a 12-month period.	LCPLTAS	To examine whether brain volumes of specific areas can predict financial capacity in aMCI patients over 1 year.	Diminishing left angular gyrus volume and right amygdala volume correlate with diminishing financial capacity.	Yes
Giannouli et al., 2021 [32]	120 patients with aMCI of the two clinical subtypes: single-domain aMCI and multiple-domain aMCI and 60 HCs. Both groups were divided into three subgroups: (a) illiterate with no formal education, (b) literate with low education, and (c) literate with high education.	LCPLTAS	To examine whether financial illiteracy in aMCI patients can negatively influence financial capacity.	Literacy has an effect on financial capacity, as the illiterate aMCI group alone had the lowest scores in LCPLTAS resembling the performance of patients with mild AD. In HCs, there is a similar pattern, but all three groups of HCs regardless of education score were above the cut-off score for financial incapacity.	Yes
Giannouli et al., 2023 [33]	35 non-institutionalized mild AD, 41 single-domain aMCI patients, 41 multiple-domain aMCI patients, and 30 HCs.	LCPLTAS	To examine the role of behavioral sleep disturbances in financial capacity.	Apart from MMSE, complex cognitive functions, such as financial capacity, are also directly linked to the frequency of sleep-disturbed behaviors both in aMCI and mild AD.	Yes
Giannouli et al., 2023 [34]	24 severe AD, 44 moderate AD, 51 mild AD, 157 aMCI, 146 HCs.	LCPLTAS	To examine whether financial altruism influences financial capacity.	More rational justification for financial choices of altruistic behavior were found in HCs and aMCI patients compared to AD patients.	Yes
Giannouli et al., 2021 [35]	28 mild AD patients carrying the APOE e4 allele and 28 matched non-carrier patients.	LCPLTAS	To examine whether genes such as APOE e4 can influence financial capacity in AD.	The presence of the APOE ɛ4 allele does not differentiate the group of mild AD patients.	Yes
Giannouli et al., 2021 [37]	30 participants under levodopa treatment for at least two years, who met the criteria PD, 28 bvFTD, and 30 HCs.	LCPLTAS	To examine the role of apathy as well as depression in PD and FTD.	PD and FTD patients’ financial capacity performance is negatively influenced by apathy and not by depression.	Yes
Giannouli et al., 2023 [38]	109 community-dwelling HCs.	LCPLTAS	To examine the role of perceived frailty on financial capacity.	Greek older women report higher frailty and higher perceived financial exploitation.	Yes
Giannouli et al., 2024 [39]	80 aMCI patients and healthy controls.	LCPLTAS	To examine the role of handedness on financial capacity.	Left-handed aMCI women scored lower in most financial capacity domains compared to right-handed men and women.	Yes
Giannouli et al., 2024 [36]	41 patients with moderate AD.	LCPLTAS	To examine the role of amyloid β peptide 1–42 (Aβ42), total tau, and phosphorylated tau on financial capacity.	No influence of these three CSF biomarkers on financial capacity.	Yes

## Data Availability

Data is contained within the article.
